# Advances in Magnetically Controlled Medical Robotics: A Review of Actuation Systems, Continuum Designs, and Clinical Prospects for Minimally Invasive Therapies

**DOI:** 10.3390/mi16050561

**Published:** 2025-05-06

**Authors:** Tiantian Kong, Qitong Zheng, Jiarong Sun, Chunxiao Wang, Huibin Liu, Zhizheng Gao, Zezheng Qiao, Wenguang Yang

**Affiliations:** 1 Shandong City Service Institute, Yantai 264005, China; 19508689023@163.com (Q.Z.); xdswxyxxb@163.com (J.S.); wangchunxiaoli888@163.com (C.W.); 2 School of Electromechanical and Automotive Engineering, Yantai University, Yantai 264005, China; cactusil@163.com (H.L.); gaozhizheng2024@163.com (Z.G.); qiaojingbiao6656@163.com (Z.Q.); yangwenguang@ytu.edu.cn (W.Y.)

**Keywords:** magnetron system, magnetic continuum, variable stiffness, low melting point alloys, medical applications

## Abstract

Magnetically controlled micro-robots hold immense potential for revolutionizing advanced medical applications, garnering significant research interest. This potential is underscored by the dual focus on magnetic control systems—both as driving forces and manipulation field sources—and magnetic continuums that have demonstrated clinical therapeutic efficacy. This comprehensive review delves into the actuation characteristics of permanent magnet systems, electromagnetic systems, and commercially available magnetic control systems. It also explores innovative designs of magnetic wires and tubes serving as continuum structures and investigates the variable stiffness properties of magnetic continua, informed by material and structural attributes. Furthermore, the discussion extends to their prospective roles and future applications within the medical realm. The objective is to elucidate emerging trends in the study of magnetic control systems and magnetic continua, marked by an expanding operational scope and enhanced precision in manipulation. By aligning these trends with clinical challenges and requirements, this review seeks to refine research trajectories, expedite practical implementations, and ultimately advocate for minimally invasive therapies. These therapies, leveraging magnetic control systems and magnetic continuums as cutting-edge treatment modalities, promise transformative impacts on the future of healthcare.

## 1. Introduction

In recent years, alongside the enhancement of living standards and the progression of medical technology, minimally invasive surgery (MIS) has garnered extensive attention. This approach offers notable benefits such as minimal invasiveness (or even non-invasiveness), heightened safety, and expedited recovery times [1,2,3]. Nonetheless, despite these advantages, the broad implementation of MIS in clinical settings encounters numerous challenges. A pivotal concern among these is the performance of driving and precise maneuvering of minimally invasive surgical instruments, which directly impacts the safety and efficacy of surgical procedures.

Minimally invasive surgical instruments are often classified as micro-robots, with the energy field employed to drive and manipulate these devices referred to as the “driving field” [4]. Presently, research has proposed various energy driving fields, encompassing optical, thermal, electric, ultrasonic, chemical, and magnetic fields [5]. Despite individual advancements in each of these fields, they each exhibit certain limitations [6]. For instance, light fields necessitate a high-intensity light source for actuation but risk irreversible harm to biological tissues due to inadequate thermal regulation [7,8,9]. Thermal fields may induce tissue inactivation or material deformation under high temperatures [10,11,12]. Electric fields face constraints in micro-robotics actuation because of electrode characteristics [13,14]. Ultrasonic fields struggle with precise motion direction control due to mechanistic and physical property limitations and may falter in bubble-containing tissues [15,16]. Chemical fields, despite offering excellent maneuverability, suffer from handling difficulties, short reaction times, and reliance on toxic fuels, restricting their broader application [17,18,19]. In contrast, the magnetic field (MF), a non-contact remote drive field, leverages magnetic torque and force to achieve precise manipulation of micro-robots by modulating flux density and magnetic field gradients [20,21,22]. Studies indicate that low-intensity magnetic fields pose no adverse effects on human tissues or material structures and can be integrated with magnetic resonance imaging (MRI) devices, enhancing their clinical viability [23,24].

**Figure 1:**
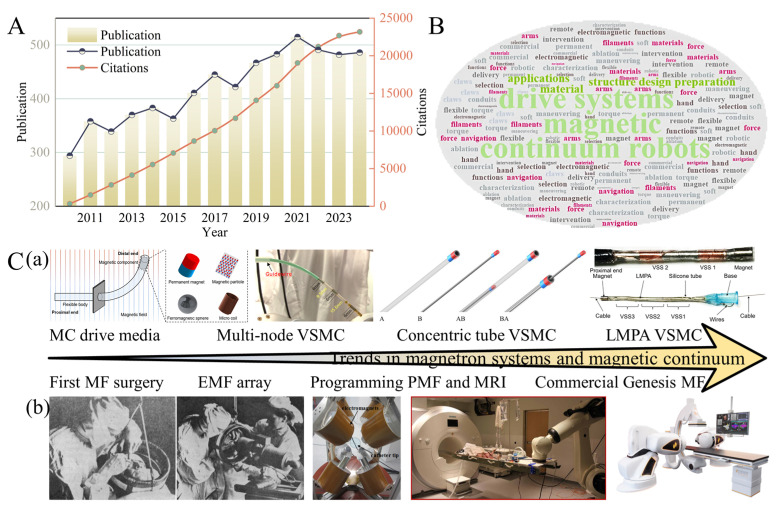
Trends in the development of magnetically actuated systems with magnetic continuums. (**A**) Research heat of magnetic drive system with magnetic continuum. (**B**) Keywords of magnetic drive system and magnetic continuum research. (**C**) Development trend and research status of (**a**) magnetic continuum and (**b**) magnetic drive system.

Magnetron systems, based on magnetic field generation principles, can be classified into three categories: permanent magnet fields (PMFs), electromagnet fields (EMFs), and commercial magnetic fields (Figure 1C(b)) [25,26,27]. The inception of utilizing an external magnetic control system for medical eye surgery dates back to the 1880s [28]. PMFs employ high-performance magnetic materials, such as neodymium–iron–boron, to create a robust gradient field with substantial magnetic force. While the magnetic continuum benefits from a blend of magnetic torque and force for operation, the flexibility of the magnetic field is challenging due to the material’s density and volume [29,30]. EMFs, on the other hand, use coil arrays that enable real-time adjustments to the magnetic field’s strength, direction, and periodicity through current signal modulation [31,32]. Although this setup offers flexible manipulation programming, it suffers from weaker magnetic fields, and the energized coils generate heat, limiting operational space [33,34]. Commercial magnetron systems, like Stereotaxis Niobe, integrate navigation technology with electromagnetic control to achieve sub-millimeter positioning accuracy clinically. However, they face ongoing challenges in coordinating multi-degree-of-freedom control and achieving system miniaturization [35,36,37].

Magnetic continuum (MC), typically constructed from guidewires or catheters integrated with magnetic materials, demonstrates flexible responsiveness to external magnetic field manipulation (Figure 1C(a)) [31,38,39,40]. Compared to conventional passive devices, these magnetic continuums, embedded with ferromagnetic segments, exhibit enhanced navigational capabilities in complex pathways. Furthermore, magnetic continua fabricated from low melting point alloys, such as Field’s metal and Bi–In–Sn composites, enable real-time stiffness adjustment through a phase transition mechanism. This addresses the challenge of balancing structural compliance and operational stability during device–tissue interactions [41,42].

**Figure 2:**
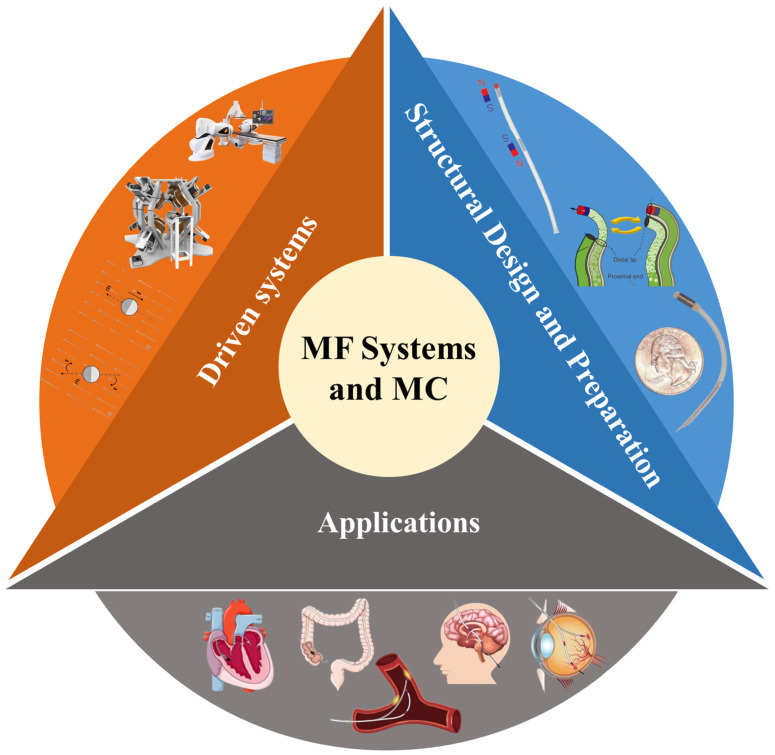
Research on magnetic drive system and magnetic continuum: magnetic driven control system, structure design and preparation, application prospect of magnetic drive system and magnetic continuum.

Magnetically actuated, magnetic continuum-based devices hold significant promise as clinical solutions for targeted therapies, cell delivery, biosensing, and the treatment of vascular diseases (Figure 2) [43,44]. Recent studies have highlighted their notable applications in vascular interventions, particularly in percutaneous coronary intervention (PCI) and atrial fibrillation (AF) ablation [45,46,47]. In PCI, a minimally invasive technique is employed to navigate a magnetic continuum through the femoral or radial arteries to address atherosclerosis-induced stenosis. This approach targets drug delivery to soften vascular rigidity and enhances arterial patency through balloon dilatation. Remarkably, this minimally invasive magnetic continuum intervention also demonstrates potential in navigating complex pathways such as cerebral nerves and vascular networks, showing considerable promise [44]. AF ablation focuses on treating cardiac arrhythmias by utilizing a transinguinal vascular approach [48]. The procedure involves the surgeon creating minor scar tissue in specific heart regions using radiofrequency heating, effectively blocking abnormal electrical signals and achieving therapeutic outcomes for AF [49].

However, most current minimally invasive clinical procedures rely on fluoroscopy (X-ray imaging), which causes radiation damage to both physicians and patients [50]. Therefore, developing a magnetic field-based magnetic continuum system could help overcome the limitations of existing technologies, optimize surgical protocols, and reduce therapeutic risks. As shown in Figure 1A, research on magnetically controlled systems and magnetic continua has become a popular field with high levels of research activity, according to relevant data from Web of Science. Unlike conventional rigid connection mechanisms, these systems enable remote non-contact control of end-effectors through magnetic fields, fundamentally overcoming the geometrical limitations of traditional mechanical transmissions [51]. As shown in Figure 1B, recent advancements in magnetic material science and electromagnetic control algorithms have driven the rapid development of two core technology directions: magnetic drive systems that combine permanent magnets and electromagnetic systems for hybrid actuation strategies to optimize energy efficiency and dynamic response [52,53]. Magnetic continuum devices are manufactured using advanced multi-material 3D printing and heterogeneous magnetization techniques. Focusing on the precise control of the magnetic continuum, we deepen the research on intelligence and motion models by optimizing the motion planning strategy of the continuum robot to achieve efficient manipulation of it [54,55]. At the same time, we explore the use of distributed artificial intelligence technology to strengthen the system’s elastic deformation control capability and enhance overall safety [56,57]. Additionally, an intelligent control framework based on machine learning offers new approaches for compensating nonlinear magnetic responses in complex environments [58].

This review systematically analyzes the technological evolution of drive systems, structural design and fabrication, and applications in terms of the progress and current status of research on permanent magnet manipulation systems, electromagnetic manipulation systems, commercial manipulation systems, conventional magnetically guided wires and magnetic catheters, and variable stiffness magnetic continuums. It critically evaluates performance limitations, summarizes recent research advances in material innovations and control methods, as well as possible applications of these recent findings in clinical medicine, and presents current challenges and future research directions. The review aims to advance the development of minimally invasive therapies based on magnetically controlled systems and the magnetic continuum, bringing them to the forefront of medical treatments.

## 2. Driven Systems

**Figure 3:**
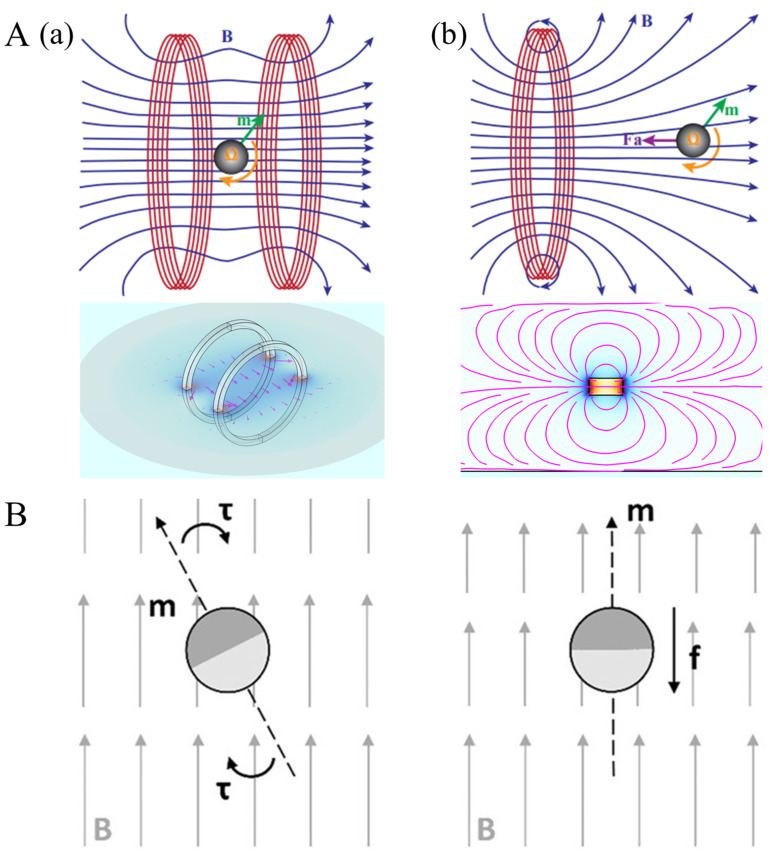
Principle of action and characterization of the magnetic drive system. (**A**), (**a**) Schematic diagram of uniform magnetic field acting through magnetic moment; (**b**) schematic diagram of gradient field acting through magnetic force and magnetic moment. (**B**) Schematic diagram of the magnetic response unit of the magnetic micro-robot subjected to the action of magnetic moment and magnetic force in the uniform field and gradient field. Reproduced from Ref. [68] with permission from Advanced Intelligent Systems.

Magnetic fields are pivotal in propelling the practical application of magnetic micro-robotics technology, serving as energy fields that drive and control micro-robots. Based on their functional characteristics, external magnetic fields can be classified into three main types: permanent magnetic fields (PMFs), electromagnetic fields (EMFs), and commercial magnetic control systems [59,60,61]. As illustrated in Figure 3A(b), a PMF generates gradient fields with varying functional properties around the permanent magnets within the external magnetic field. The magnetic response unit embedded within the magnetic micro-robot reacts to the magnetic force exerted by the gradient field and the moment aligned along the magnetic field’s direction. This mechanism allows the external magnetic field to effectively drive and control the magnetic micro-robot [62].

(1) $$F_{m}=\int_{V_{m}}\left(m\cdot \nabla \right)B{\mathrm{dV}}_{m}$$

(2) $$T_{m}=\int_{V_{m}}m\times B{\mathrm{dV}}_{m}$$

where $F_{m}$ is the magnetic force (N); $T_{m}$ is the magnetic torque in Newton meters (N-m); $V_{m}$ is the magnetization volume in cubic meters (m^3^); B is the internal magnetization strength in amperes per meter (A-m-1); m is the flux density of the magnetic field at the Tesla (T) integration point, which can also be expressed as

(3) $$B=\mu_{0}H$$

where $\mu_{0}$ is the permeability of free space, is $4\pi \times 10^{-7}T\cdot m\cdot A^{-1}$; and H is the field strength in amperes/meter. When the overall size of the magnetic object varies spatially with respect to the external magnetic field, the above relationship can be simplified as follows

(4) $$F_{m}=\left(M\cdot \nabla \right)B$$

(5) $$T_{m}=M\times B$$

where M is the total dipole moment of the controlled object in amperes, and can be simplified with Maxwell’s equations, Equations (4) and (5), and can be expressed as

(6) $$F_{m}={\left[\begin{array}{ccc} \frac{\partial B}{\partial x} & \frac{\partial B}{\partial y} & \frac{\partial B}{\partial z} \end{array}\right]}^{T}M=\left[\begin{array}{ccc} \frac{\partial B_{x}}{\partial x} & \frac{\partial B_{x}}{\partial y} & \frac{\partial B_{x}}{\partial z}\\ \frac{\partial B_{x}}{\partial y} & \frac{\partial B_{y}}{\partial y} & \frac{\partial B_{y}}{\partial x}\\ \frac{\partial B_{x}}{\partial z} & \frac{\partial B_{y}}{\partial z} & -\left(\frac{\partial B_{x}}{\partial x}+\frac{\partial B_{y}}{\partial y}\right) \end{array}\right]$$

(7) $$T_{m}=\left[\begin{array}{ccc} 0 & B_{z} & -B_{y}\\ -B_{z} & 0 & B_{x}\\ B_{y} & -B_{x} & 0 \end{array}\right]\left[\begin{array}{c} M_{x}\\ M_{y}\\ M_{z} \end{array}\right]$$

This relationship offers an intuitive explanation: magnetism only manifests in the presence of a magnetic field gradient, which tends to move the object toward the location of the local maximum magnetic field strength [63,64,65,66]. A magnetic moment arises when there is an angular difference between the object’s magnetization and the applied magnetic field, leading to alignment of the object’s magnetization with the magnetic field.

As shown in Figure 3A(a), an electromagnetic field can generate a uniformly distributed magnetic field within the working area [67]. In such a uniform magnetic field, the magnetic response unit of the micro-robot undergoes twisting deformation due to torque. By combining this behavior with programmable changes in the direction of the magnetic field and the design structure of the micro-robot, precise control of its motion and operation is achieved. As shown in Figure 3B, commercial magnetic control systems typically integrate PMFs and EMFs to generate magnetic force using gradient fields and torque using uniform fields, enabling flexible and precise manipulation of magnetic micro-robots [68].

Magnetic control systems, as core devices for driving and controlling magnetic continua, hold significant research value in the field of minimally invasive therapy and medical robotics. This section analyzes the drive characteristics of permanent magnet systems, electromagnetic systems, and commercial magnetic control systems, evaluates their advantages and limitations in medical applications, and explores future trends based on current research.

### 2.1. Permanent Magnet Control System

**Figure 4:**
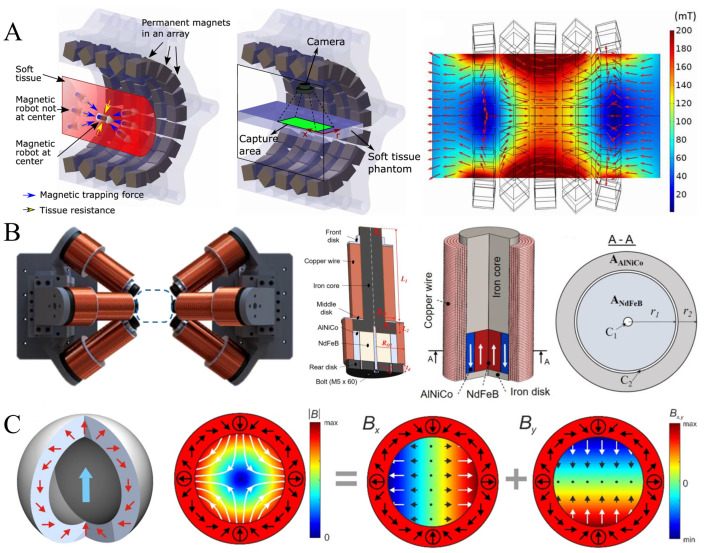
State of the art of research on permanent magnet maneuvering systems. (**A**) Permanent magnet array-based drive manipulation system. Reproduced from Ref. [73] with permission from Science Advances. (**B**) Electro–permanent magnet composite magnetic control system. Reproduced from Ref. [74] with permission from International Journal of Mechanical Sciences. (**C**) Halbach Cylindrical Optimum Magnetic Control. Reproduced from Ref. [75] with permission from Cells.

The permanent magnet system (PMF) is a non-contact drive device based on permanent magnetic materials. Its core principle involves using high-performance magnetic materials, such as neodymium–iron–boron and samarium–cobalt, to generate a stable magnetic field. By adjusting the magnetic flux density and magnetic field gradient, it controls the micro-robot [69,70]. Because PMFs do not require an external power source and have a simple structure, they are highly appealing for medical applications. Additionally, PMFs typically exhibit strong magnetic field strength and can provide high drive torque, making them excellent for channel-based operations like navigating through blood vessels or cavities. For instance, in closed channels, the combination of magnetic torque and magnetic force enables efficient propulsion of micro-robots. Furthermore, permanent magnetic fields offer a stable magnetic field, suitable for long-duration tasks [71,72]. Son et al. proposed a permanent magnet array-based continuous drive manipulation system (Figure 4A) to address the problem of limited self-propulsion and power supply capabilities of micro-robots. Driven by this system, it is possible to make millimeter-scale magnetic micro-robots traditionally move freely in biological tissues to perform operations such as biopsy, hyperthermia, and cauterization [73]. In addition, Nguyen et al. designed a composite electro–permanent magnet drive system for micro-robot manipulation by combining permanent magnets with electromagnets (Figure 4B). This system combines the advantages of the larger magnetic field strength of conventional permanent magnets and the programmable manipulation of electromagnets, and, with a maximum gradient field and magnetic field of 1.9 T/m and 124 mT, the total enhancement is about up to 30% [74]. Blümler et al. have explored in depth the possible applications of permanent magnet systems to manipulate nanoparticles and cells based on permanent magnets with adjustable magnetic fields (Figure 4C). By evaluating the magnetic field strength of the permanent magnets, the size of the device, and the possibility of arrays, it was concluded that the Halbach cylinder might be the optimal magnetic control device [75].

In recent years, numerous studies have concentrated on the optimal design of permanent magnetic systems and their healthcare applications. Mahoney et al. combined the practical operation of a magnetic capsule endoscopic micro-robot with a mobile or stationary device featuring eight independently controllable degrees of freedom (DOF), utilizing seven specifically distributed permanent magnets [76]. This approach resolved the limitations of requiring specific rotational manipulation and visualization when driving with a single rotating permanent magnet, achieving multi-agent and complex mechanism motions [77]. Similarly, Salmanipour et al. developed a system demonstrating a mobile or stationary device with up to eight independently controllable degrees of freedom using seven specifically distributed permanent magnets for multi-agent or complex mechanism motion [78]. Khalil et al. utilized an open-architecture robotic system with a neodymium–iron–boron magnet mounted on the end-effector of a robotic arm, creating a closed-loop control system for drug delivery applications by plotting the relationship between the position of microparticles and the configuration of the robotic arm. Further advancing open-structured systems for controlling the magnetism of drug carriers is crucial for in vivo application research [79]. Pittiglio et al. addressed the motion control problem of magnetic capsule endoscopes by proposing an innovative actuation scheme to counteract gravity’s effects and enable non-planar lumen inspection. The method applies force and torque on the internal magnetic capsule via an external magnet, with precise control facilitated by a serial manipulator [80]. To optimize performance, a control strategy based on a nonlinear inverse stepping method was introduced, reducing contact with the colon wall and, thus, friction. Experimental results showed this method significantly reduces the collision probability between the capsule robot and lumen structures, providing reliable technology for non-planar lumen inspection. Pittiglio et al. also proposed an innovative large workspace building scheme by synergistically controlling the internal micro-device through an external magnetic field and magnetic field gradient using two permanent magnets [40]. Theoretical and experimental validation indicated this method adds up to three degrees of freedom to magneto mechanical manipulation, with a maximum crosstalk value of 6.1% and an average error of 11.1% in practical applications [81]. Ryan et al. achieved significant breakthroughs in studying magnetically actuated micro-robots for biomedical and microfluidic applications by using an array of rotating permanent magnets to precisely control a tetherless micrometer-scale device [82]. They optimized the magnetic field distribution to reduce system energy consumption, covering a magnetic field range from 0 to 30 mT and a gradient strength interval from 0 to 0.83 T/m, with extremely high operational precision (spatial resolution of 25 μm, average error of 250 μm) [83].

Permanent magnet systems hold promising applications in the medical field, especially in vascular interventions. However, they also have limitations: first, their magnetic field strength is limited by the volume and density of the magnetic material, and a larger magnetic field requires a larger device volume; second, the magnetic field of PM systems cannot be turned off or adjusted in real-time, limiting flexibility; and lastly, their manipulation capability is restricted in complex operating environments, such as unstructured pathways [84,85]. To enhance flexibility and applicability, future research should focus on developing new magnetic materials with high magnetic properties, optimizing magnetic field distribution to improve operational efficiency, and exploring hybrid strategies between PM systems and other drive modes, such as electromagnetic systems [86,87].

### 2.2. Electromagnet Control System

Electromagnet systems (EMS) leverage coil arrays to dynamically modulate magnetic field strength, orientation, and periodicity through real-time adjustments of electrical current signals—encompassing amplitude, waveform, and frequency [88]. This inherent programmability confers upon EMS a superior adaptability for dynamic tasks compared to their permanent magnet counterparts, rendering them particularly suited for precision-oriented applications such as targeted drug delivery or cellular therapy. When coupled with sophisticated navigation modalities like MRI, EMS can attain sub-millimeter positioning accuracy, thereby augmenting their clinical utility.

**Figure 5:**
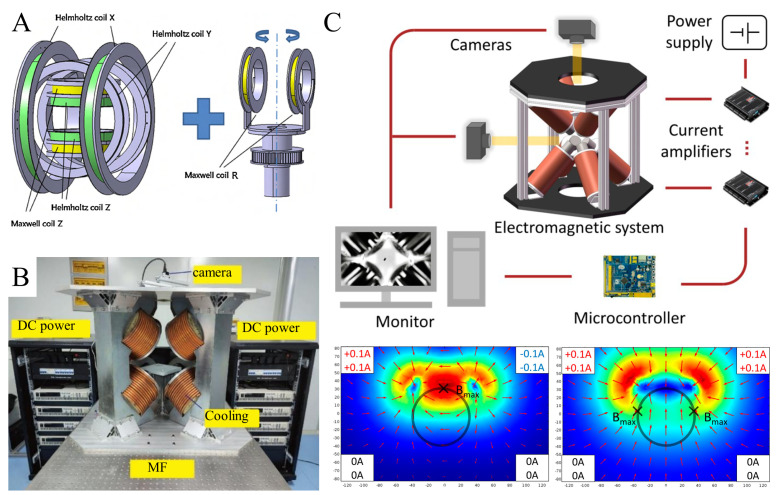
Current status of research on electromagnet manipulation systems. (**A**) A mixed action system of Helmholtz and Maxwell coils. Reproduced from Ref. [89] with permission from Sensors and Actuators A: Physical. (**B**) An 8-coil electromagnetic system with 100 mT and 300 mm diameter workspace built using the array method. Reproduced from Ref. [40] with permission from Magnetochemistry. (**C**) Multi-polar three-dimensional electromagnetic system with large working space. Reproduced from Ref. [92] with permission from Micromachines.

In recent times, extensive research has been dedicated to refining the performance characteristics of electromagnetic systems and expanding their healthcare applications. Yu et al. developed a novel EMA system (Figure 5A) using four fixed coil pairs and one rotating coil pair in order to improve the small magnetic field strength and high heat generation of electromagnetic coils. Driven by this EMA system, the Helmholtz coils generate a magnetic torque that can be used to align the magnetized micro-robot, and the Maxwell coils provide a gradient field that can be used to provide propulsion [89].To overcome spatial limitations inherent in magnetic field operational zones, Petruska et al. introduced distributed electromagnet configurations distinguished by variable coil counts and arrangements [60], each offering distinct benefits across torque management, force output, patient compatibility, imaging integration, scalability, and energy efficiency. Choi et al., aiming at improving the compactness of external magnetic field actuators, put forth a streamlined EMS design incorporating paired Helmholtz and Maxwell coils [90], which merges the advantageous traits of both coil types to achieve reduced dimensions alongside enhanced electromagnetic properties. Lin et al., seeking to expand operational capacity while maintaining high magnetic field intensity, employed an 8-coil array configuration (Figure 5B) [40], enabling the generation of a 100 mT magnetic field within a 300 mm diameter workspace. Chen et al.’s 5-coil EMS design [48] strikes a balance between uniform and gradient magnetic fields, enhancing portability and application versatility via structural simplification. Wang et al., with an emphasis on clinical steering tasks, devised an EM system integrated with three serial robotic arms for advanced navigation [91], significantly broadening operational reach and synergy with imaging technologies. Li et al. used a multi-stage electromagnet to build a three-dimensional magnetic electromagnetic system with a large operating space that could drive nickel nanoparticles to flexibly translate and rotate with a maneuverability of 15 mm/s and 3 Hz. (Figure 5C). The electromagnetic system consists of eight coils arrayed in a cube and is capable of obtaining a spherical workspace with a diameter of 100 mm and a magnetic field with a maximum of about 4 mT [92].

Despite their considerable potential in executing complex medical tasks dynamically, EM systems face certain constraints: inadequate magnetic field strength for high-torque requirements, thermal generation within energized coils that may compromise long-term stability, and the bulkiness of devices coupled with restricted operational areas, which hinder miniaturization efforts [93,94]. Future advancements should therefore concentrate on developing energy-efficient, low-heat electromagnetic drivers; fine-tuning magnetic field distributions for heightened precision; and investigating hybrid propulsion mechanisms that marry electromagnetic systems with alternative drive modalities, such as permanent magnet systems [95,96]. These endeavors will be pivotal in unlocking the full clinical potential of electromagnet systems.

### 2.3. Commercial Magnetic Control Systems

Commercial magnetron systems stand as sophisticated embodiments of technology, seamlessly merging avant-garde navigation capabilities with sophisticated electromagnetic control algorithms, thereby finding extensive resonance within clinical environments. A case in point is the array of systems meticulously crafted by Stereotaxis, Inc., meticulously engineered with an unwavering commitment to facilitating high-precision, minimally invasive therapeutic interventions [97]. These systems boast robust magnetic fields, exerting substantial driving torques crucial for the meticulous manipulation of magnetized wires and catheters in clinical scenarios, notably in percutaneous coronary interventions (PCI), where they adeptly navigate magnetized catheters to execute tasks such as balloon dilation or localized drug delivery at stenotic sites.

**Figure 6:**
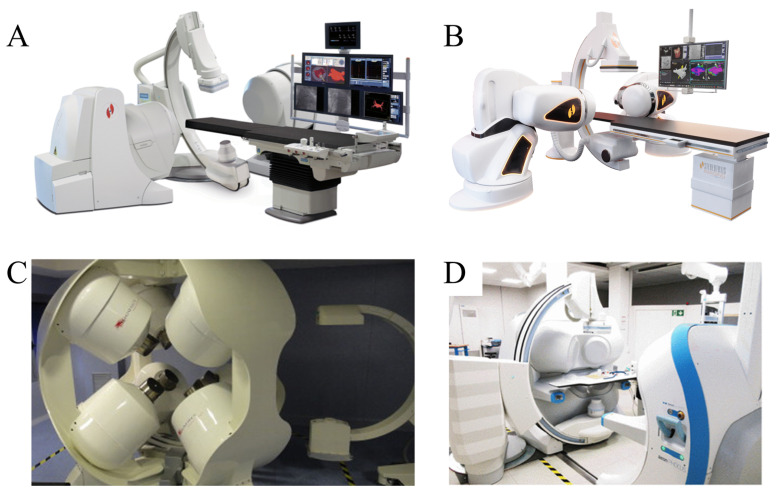
Current state of research on commercial manipulation systems. (**A**) Niobe platform. Reproduced from Ref. [98] with permission from Journal of Cardiovascular Electrophysiology. (**B**) Genesis system. Reproduced with permission from Stereotaxis, Inc. (**C**) CGCI system. Reproduced with permission from Neuro-Kinesis. (**D**) Aeon Phocus system. Reproduced with permission from ETH Zurich.

Recent strides in this domain have been marked by an unrelenting pursuit to enhance the performance and broaden the clinical utility of these magnetic control systems. At the vanguard of this innovation is Missouri’s Stereotaxis, which, in 2003, unveiled the groundbreaking Niobe platform (depicted in Figure 6A), featuring a unique dual-focused permanent magnet architecture [98]. This ingeniously designed permanent magnet unit, affixed to the distal extremity of a specialized robotic arm, enables precise vectorial command over the central magnetic field through rotational and tilting motions of the magnet housing. Initially conceived for catheter guidance in cardiac arrhythmia therapies, its influence has since proliferated across diverse medical disciplines, having facilitated over 130,000 clinical interventions globally. The subsequent Genesis System (Figure 6B) represents a leap forward, achieving a 70–80% boost in operational velocity through magnet miniaturization and kinematic refinement. Its novel magnet design permits central point rotation and, coupled with a streamlined profile, markedly enhances spatial efficiency during patient procedures. In addition, California’s Magnetics has also made significant contributions with the development of the Catheter Guidance Control and Imaging (CGCI) system (Figure 6C), employing an intricate eight-pole electromagnetic array configuration comprising four upper and four lower units, tailored specifically for creating a closed-loop magnetic field ideal for catheter ablation procedures. However, its fully encapsulated design imposes spatial limitations during surgical operations. Conversely, Aeon Scientific AG from Zurich, Switzerland, introduces the Phocus system (Figure 6D), adopting a dual-frame, seven-pole electromagnetic schema that balances maintaining formidable magnetic field strength with preserving vital space for intraoperative imaging and surgical maneuvers through its detachable bilateral structure [99]. Furthermore, Smith et al. have proposed an augmented strategy rooted in the Stereotaxis Niobe platform, leveraging machine learning algorithms to navigate intricate vascular pathways with pinpoint accuracy [100].

Despite their indisputable value in clinical contexts, these commercial magnetic control systems grapple with several constraints: their considerable dimensions pose obstacles to miniaturization and portability aspirations; the intricacies of coordinating multi-degree-of-freedom controls present technical hurdles, thereby restricting their deployment in more complex procedural landscapes; and prohibitive costs impede their widespread adoption. To surmount these challenges and propel clinical applications forward, future research endeavors should concentrate on cultivating more compact and portable commercial systems, refining multi-degree-of-freedom control algorithms to augment operational efficacy, and exploring synergistic hybrid approaches that intelligently merge commercial systems with complementary actuation modalities, be it permanent or electromagnetic systems.

This chapter presents a comprehensive analysis of the operational characteristics of permanent magnet manipulator systems, electromagnet manipulator systems, and commercially available magnetron systems within the realm of healthcare applications. As shown in the comparative analysis of magnetic drive systems in Table 1, findings illustrate that each system boasts distinct advantages alongside inherent limitations: the permanent magnet system excels in facilitating high-precision navigation during channel-based procedures; the electromagnet system demonstrates superiority in dynamic adjustments and multi-degree-of-freedom control; while the commercial system shines in clinical validation and practical implementation [101]. Future research endeavors must aim to refine the performance and broaden the applicability of magnetic control systems by addressing the specific challenges and requirements of medical treatments. This includes the exploration of novel magnetic materials, enhancement of magnetic field distribution, and the investigation of hybrid propulsion methodologies. Such advancements will serve to propel minimally invasive therapeutic techniques, grounded in magnetron systems, towards unparalleled levels of efficiency and precision [102].

## 3. Structural Design and Preparation of Magnetic Continua

The notion of magnetically steerable catheterization finds its origins in the 1950s, when Tillander pioneered an endeavor to affix a diminutive steel chain to a catheter’s extremity and ventured into magnetic guidance utilizing electromagnets [106]. This groundbreaking effort laid the foundation for subsequent explorations, as numerous research groups, from the 1960s through the 1980s, delved into the creation of a diverse array of magnetically guided continuous devices, propelling technological advancements within the domain. Concurrently, magnetic continua, integral to the fabric of magnetically controlled micro-robotic systems, have exhibited a broad spectrum of applications across medical therapy, industrial examination, and micromanipulation disciplines [107,108]. At their core, these systems hinge upon the dynamic modulation of material rigidity and conformation via external magnetic fields, enabling them to adeptly navigate intricate and evolving operational landscapes [109].

In this section, our focus lies with elucidating the design characteristics of magnetic guidewires and magnetic conduit continua, predicated on permanent magnet configurations. We will meticulously examine the practical implementations of these innovative constructs within the medical arena and extrapolate their prospective trajectories, informed by the latest strides in research pertaining to variable stiffness magnetic continua derived from low melting point alloy technologies. This in-depth analysis aims to shed light on how these advancements are poised to shape the future of minimally invasive therapeutic interventions.

### 3.1. Continuum of Magnetically Guided Wires and Magnetic Conduits

Magnetic guides (MGs) and magnetic conduits (MCs) stand as pivotal research focal points within the realm of magnetically continuous robotics. These devices, typically comprising a ferromagnetic material layer that engenders a magnetic response and an elastomer subject to deformation under the influence of external magnetic fields, possess a remarkable capacity to navigate and maneuver through intricate environments. This characteristic renders them invaluable in medical contexts, particularly in vascular interventions and endoscopic procedures, where precision and adaptability are paramount [68,110].

The efficacy of the magnetic catheter design is fundamentally rooted in meticulous material selection and structural optimization. Frequently constructed from ferromagnetic alloys such as nickel–titanium (NiTi) or iron-based materials, these devices integrate magnetic particles to augment their responsiveness to external magnetic stimuli. Research has illuminated that parameters including stiffness, bending characteristics, and sensitivity to external magnetic fields are pivotal determinants of operational accuracy for MGs [111]. For instance, refining the material composition and microstructure holds the potential to substantially enhance the flexibility of magnetic catheters when traversing constricted or meandering vascular terrains. The design intricacy of magnetic catheters often necessitates a multilayered architecture, incorporating a ferromagnetic core, an insulating intermediary, and a protective outer sheath. This stratified design not only bolsters their manipulative versatility but also reinforces their resilience amidst the rigors of complex operational milieus [102]. By leveraging such advanced designs, magnetic catheters can potentially elevate the precision and safety of minimally invasive medical interventions, ushering in a new era of therapeutic refinement.

**Figure 7:**
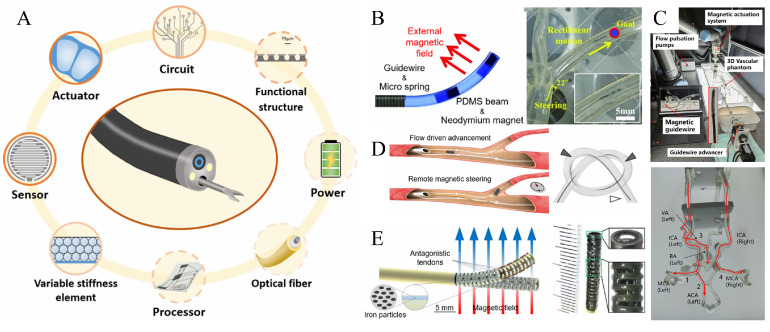
Status of research on the magnetic guidewire and catheter continuum. (**A**) Critical and scalable technologies for magnetic guidewire and conduit robotics. Reproduced from Ref. [112]. with permission from Advanced Intelligent Systems. (**B**) Magnetically guided filaments composed of a bundle of silica gel (PDMS), two NdFeBs, and a connected microspring. Reproduced from Ref. [119] with permission from Soft Robotics. (**C**) Magnetically controlled guidewire robotic system (MCGRS) with steering and propulsion capabilities. Reproduced from Ref. [115] with permission from Advanced Intelligent Systems. (**D**) Tethered ultra-flexible endovascular robot driven by blood flow. Reproduced from Ref. [116] with permission from Nature Communications. (**E**) Hybrid CR synthesized from two drive mechanisms. Reproduced from Ref. [117] with permission from Advanced Intelligent Systems.

Recent research strides have centered on enhancing the design intricacy and clinical applicability of MGs and MCs. A comprehensive review by Zhu et al. encapsulated pivotal technologies and scalable innovations across actuators, circuitry, functional architecture, power systems, optical fibers, processors, variable stiffness elements, and sensor technology (depicted in Figure 7A), underscoring the multifaceted nature of advancements in this domain [112]. In translating these innovations to practical applications, a delicate equilibrium must be struck between device attributes—such as reusability versus single-use—and the demands of diverse clinical scenarios. This balancing act encompasses considerations of cost, automation levels, durability, efficiency, adaptability, material availability, and quality consistency. Novel and refined design methodologies have thus emerged to address these complexities. Kim et al., for instance, put forth a sub-millimeter ferromagnetic soft continuum robot, ingeniously synthesizing magnetic micro/nanoparticles within a polymer matrix to fashion a steerable elastomer [113]. This process entails magnetizing an uncured blend under a pulsed magnetic field, followed by shaping via advanced manufacturing techniques like 3D printing, extrusion, or injection molding. The accompanying system, comprising a custom magnetic conduit, robotic arm, motorized linear drive, and remote-control unit, underwent rigorous in vitro and in vivo navigation trials within a realistic human neurovascular phantom and porcine brachial artery, affirming its negotiation process through convoluted pathways. Soft materials, particularly elastomers and hydrogels, have garnered attention for their exceptional elasticity and malleability, rendering them prime candidates for constructing highly maneuverable tips. Yang et al.’s development of a ferromagnetic MG with a core–shell configuration—featuring a rigid shaft paired with a pliable tip—epitomizes this balance, ensuring efficient steering without compromising tip integrity during operations [114]. Furthermore, the integration of ultrasound imaging facilitates real-time intervention monitoring. Addressing the dual imperatives of minimizing physician radiation exposure and harnessing the potential of magnetic MG systems in vascular procedures, Fu et al. introduced the magnetically controlled guidewire robotic system (MCGRS). This system, grounded in sophisticated modeling and trajectory planning (Figure 7C), employs constant curvature theory alongside a synergistic dipole-Cosserat rod model to forecast tip deformation accurately [115]. Its efficacy was validated through additional navigation tests conducted in a meticulously crafted 3D vascular replica. Hybrid actuation mechanisms, merging magnetic actuation with alternative modalities, present avenues for leveraging each system’s distinct kinematic attributes to elevate overall performance. Pancaldi et al.’s tethered ultraflexible endovascular robot, propelled by blood flow dynamics (Figure 7D), exemplifies this concept [116]. Comprised of a μ-catheter and magnetic head, its locomotion hinges on hydrodynamic forces, with magnetic actuation facilitating nuanced steering at vascular bifurcations. Both computational simulations and in vivo rabbit ear experiments attest to its capability to navigate intricate vascular networks with minimal external guidance, albeit with limitations in compromised blood flow conditions.

Zhang et al.’s hybrid magnetic catheter robot (CR) represents another innovative stride, integrating a dual-drive mechanism for expansive steering capabilities (up to 100°) and precise manipulations (finer than 2 μm) (Figure 7E) [117]. Its core structure, a 3D-printed manipulator with an ultrathin hollow skeleton, is augmented with a ferromagnetic elastomer coating, marrying tendon-driven and magnetic-driven strategies seamlessly. Peyron et al.’s magnetic concentric tube robot stands out for its dexterous yet stable design, achieved through a assembly of pre-bent or straight tubes and strategically positioned magnetic elements [118]. Boasting a remarkable minimum distal radius of curvature (5.4 mm) and a broad angular displacement range (up to 160°), all while maintaining a sub-millimeter outer diameter, it epitomizes precision engineering. Jeon et al.’s silicone (PDMS)-based magnetic guide wire system (Figure 7B) integrates a NdFeB magnet, micro spring, and silica gel material at its tip, complemented by a finite element analysis-derived mathematical model for precise deformation prediction [119]. Experimental outcomes revealed impressive angular deformities exceeding 130°, coupled with stellar performance across 2D and 3D vascular models. Wang et al.’s model-centric evolutionary design approach marries theoretical modeling with genetic algorithms, targeting optimization of catheter magnetization patterns and rigidity characteristics [120]. This strategy has proven instrumental in expanding the operational envelope of catheters and offers a potent optimization tool for future magnetically controlled robot (MCR) designs. Lloyd et al.’s multi-segment continuous magnetic device, composed of pure and magnetic Ecoflex segments, adopts a neural network training methodology informed by finite element analysis to enable patient-specific customization. This personalized design paradigm ushers in a new era of precision in medical robotics. Liu et al.’s robotic catheter system with tunable steering performance breaks away from conventional soft material deformation segments, instead utilizing a heat-responsive tube structure capable of multiple flexion modes [121,122]. Through meticulous multi-physics field simulations and iterative algorithms, they uncovered the pivotal role of flexion modes in dictating deformation behavior and steering responsiveness, thereby advancing our understanding of key design parameters.

While magnetic guidewires and catheters have shown great promise in the medical field, current designs face several challenges that limit their clinical utility. Firstly, there is a restricted range in adjusting stiffness, which hinders their ability to perform complex clinical tasks effectively. Secondly, the optimization of manipulation precision through the strength and distribution of external magnetic fields has yet to reach its full potential. Lastly, the performance of magnetic materials can degrade significantly under extreme environmental conditions, such as high temperatures or humidity [123]. Future research should focus on several key directions to overcome these limitations. Developing new ferromagnetic materials with enhanced magnetic field responsiveness could lead to more efficient devices. Improving structural design methodologies will enable a broader range of stiffness adjustments, thereby enhancing versatility in clinical applications. Additionally, integrating multi-physical field simulation techniques will further refine manipulation accuracy, ensuring these devices can meet the demands of intricate clinical scenarios.

### 3.2. Variable Stiffness Magnetic Continuum Based on Low Melting Point Alloys

Variable stiffness magnetic continuous robots (VS-MCRs) based on low melting point alloys (LMPAs) represent a novel class of magnetic materials that dynamically modulate their stiffness in response to external stimuli, such as magnetic fields and temperature variations [123]. These systems hold significant promise for medical applications, particularly in scenarios demanding precise manipulation and adaptation to complex operating environments. The fundamental principle underlying these robots is the phase transition characteristics of LMPAs, like Field’s metal or Bi–In–Sn alloys, which undergo a solid-to-liquid transformation within a specific temperature range. Leveraging this property, researchers have engineered systems capable of toggling between soft and hard states in the presence of an external magnetic field, facilitating dynamic stiffness adjustments [124]. For instance, one study explored continuous robots fabricated from Bi–In–Sn alloys, achieving rapid phase transitions in response to external cues [125]. Experimental outcomes demonstrated that the robot could swiftly shift from a soft to a stiff state under the influence of an external magnetic field in less than one second. This capability substantially enhances the robot’s adaptability to intricate operating conditions, thereby broadening its potential applications in precision medicine and minimally invasive procedures.

**Figure 8:**
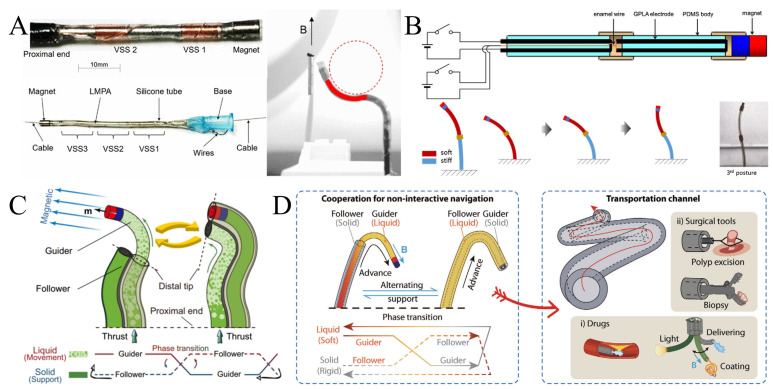
State of the art of research on variable stiffness magnetic continuums based on low melting point alloys. (**A**) Variable stiffness (VS) magnetic conduit robot composed of two and three variable stiffness segments (VSS). Reproduced from Ref. [31] with permission from Advanced Intelligent Systems. (**B**) Robot with variable stiffness properties realized by phase transition of graphene polylactic acid (GPLA). Reproduced from Ref. [126] with permission from Sensors and Actuators A: Physical. (**C**) Variable-stiffness magnetic continuum robot with a stable solid-state skeleton and for propulsion. Reproduced from Ref. [127] with permission from Nature Communications. (**D**) Continuum robot consisting of coaxially assembled Guider and Follower components. Reproduced from Ref. [128] with permission from Advanced Functional Materials.

Recent research has increasingly focused on integrating low melting point alloys (LMPAs) into magnetic continuum robots for medical applications. Chautems et al., for example, designed prototypes for cardiac ablation and gastrointestinal endoscopy using LMPA-based variable stiffness (VS) magnetic catheter robots with two or three variable stiffness segments (VSS) (Figure 8A) [31]. Their process involves injecting melted LMPA into a silicone tube placed on a heated plate to create cylindrical variable stiffness magnetic conductors. Conductive copper wires are wrapped around the LMPA, enabling the programming and control of guidewire stiffness. This design allows for multi-radius bending using a single permanent magnet, which is particularly useful in applications such as catheter ablation and gastrointestinal endoscopy [6]. Park et al. introduced a magnetic manipulator capable of magnetic steering via a permanent magnet at its tip and variable stiffness through a phase change in graphene-poly(lactic acid) (GPLA) (Figure 8B) [126]. By designing a multi-node circuit to programmatically control the temperature and state of the LMPA, they achieved flexible manipulation of the magnetic gripper. Experimental validation showed that the two-segment body of the robot could achieve large-angle deformation. Cao et al. developed a novel magnetic programmable continuous robot (MMPCR) with three different deformation modes—J-shape, C-shape, and S-shape—based on a variable stiffness alloy [63]. The deformation direction and curvature of the robot can be adjusted according to specific needs. Experimental results indicated an average angular deviation error of 3.3°, highlighting its high precision and flexibility. Zhang et al. presented a novel VS-MCR for endoscopic surgery, which can adjust its stiffness by changing the catheter length or inducing the solid-liquid phase transition in LMPAs [127]. They also proposed a quasi-static stiffness model and a learning-based stiffness compensation method to accurately estimate the stiffness of the robot. Experimental results demonstrated that the VS-MCR significantly enhances resistance to external disturbances and prevents tip deformation, with an average positional deviation of only 1.1 mm. These advancements underscore the potential of variable-stiffness magnetic continuum robots for medical applications, especially in tasks requiring precise navigation and adaptation to complex environments [5]. Mao et al. developed a millimeter-scale continuum robotic system based on the phase-transition properties of a low-melting-point alloy (Figure 8C) [127]. The device cleverly combines a “guide” and “follower” structural design to achieve apical extension while maintaining structural stability. Precisely controlled by a programmable magnetic field, the robot is capable of performing tip-based elongation cycles: each cycle consists of a solid skeleton phase for stabilizing support and a liquid component phase for propulsion, enabling autonomous shaping independent of environmental interactions [4]. Additionally, the study demonstrated the device’s success in transporting microsurgical tools into a tortuous and fragile canal environment, in conjunction with clinical imaging techniques. Yang et al. further optimized the overall structure to design an integrated and miniaturized continuous robotic system (Figure 8D) [128]. The system comprises coaxially assembled Guider and Follower components, utilizing phase change materials for non-interactive navigation and multi-functional operation. The navigation process is achieved through alternating softening–hardening cycles: first, the Guider softens and is magnetically guided while the rigid Follower provides structural support; subsequently, the Follower softens to replicate the path while the Guider solidifies to form a new skeleton. This mechanism not only maintains structural integrity but also allows navigation along a predetermined trajectory, significantly reducing interactions with the cavity [129]. Moreover, the group further developed a continuous robotic system consisting of three independent units, which realized precise control of multi-degree-of-freedom motions through segmental stiffness adjustment. This system successfully performed multifunctional operations such as directional conveying, precise painting, grasping, and obstacle removal.

Variable stiffness magnetic continuum robots utilizing low melting point alloys (LMPAs) have demonstrated substantial research potential in the medical field [129]. However, current studies face several limitations: firstly, the phase transition process is highly sensitive to temperature fluctuations, which may compromise stability during practical applications. Secondly, the high cost of alloy materials restricts their widespread clinical use. Lastly, the performance of these alloys can significantly degrade under extreme environmental conditions, such as high temperatures or high humidity [130]. Future research should focus on the following areas: developing new low-melting-point alloy materials to enhance the response speed and reliability of the phase transition; optimizing the heating and cooling processes to improve the system’s temperature stability; and integrating multi-physics field simulations to increase the accuracy of stiffness adjustments.

This section provides a systematic analysis of the design characteristics of magnetically guided wires and magnetic conduit continuums, as well as the progress in research on variable stiffness magnetic continuums based on low melting point alloys. As shown in Table 2, a comparative analysis of magnetically wires, conduits, with variable stiffness magnetic continuums, the findings indicate that these designs have significant applications in the medical field. However, to further enhance their performance and applicability, future research must address the pain points and specific needs of medical treatments, clarify research directions, and promote the application of research outcomes. Looking ahead, advancements in material science and control algorithms are expected to make minimally invasive therapeutic techniques based on magnetron systems and magnetic continuums a cornerstone of future advanced treatment methods. Through continuous improvements in design and performance, and by closely aligning with clinical needs, these magnetic continuum systems are poised to become a vital component of future advanced medical technologies, potentially revolutionizing the field of minimally invasive therapies.

## 4. Applications

**Figure 9:**
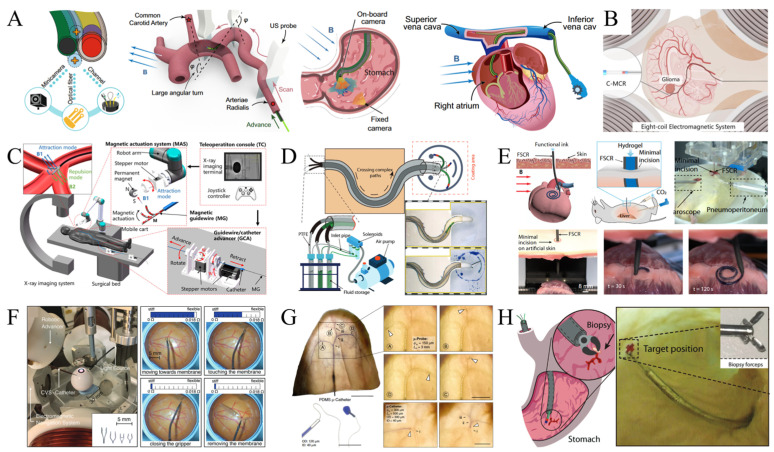
Application prospects of magnetically actuated systems with magnetic continuums. (**A**) Specific applications of magnetically driven systems and magnetic continuums in targeted therapy and drug delivery, cell delivery and biosensing, vascular interventions, and neurosurgical treatments. Reproduced from Ref. [127] with permission from Nature Communications. (**B**) With four modes of operation, it is capable of navigating complex pathways and delivering nanomedicines over long distances to specific regions through its internal channels. Reproduced from Ref. [40] with permission from Magnetochemistry. (**C**) Magnetically controlled robotic system for interventional procedures. Reproduced from Ref. [115] with permission from Advanced Intelligent Systems. (**D**) Coaxially assembled “Guider” and “Follower” magnetic continuum robotic operating tips draw predefined shapes on a 2D plane. Reproduced from Ref. [128] with permission from Advanced Functional Materials. (**E**) Minimally invasive in situ computer-controlled bioprinting of conductive hydrogels on pig tissue surfaces by a ferromagnetic flexible catheter robot. Reproduced from Ref. [139] with permission from Nature Communications. (**F**) Robotic retinal epiretinal detachment surgery in an eye model. Reproduced from Ref. [51] with permission from Advanced Science. (**G**) Tethered ultraflexible endovascular microprobe utilizes hydrokinetic energy to transport through a tortuous vascular network with minimal external intervention. Reproduced from Ref. [116] with permission from Nature Communications. (**H**) The Guider and Follower magnetic continuum robots manipulate biopsy forceps for sampling in the stomach. Reproduced from Ref. [127] with permission from Nature Communications.

The study of magnetic drive control systems and magnetic continuums holds significant theoretical importance and demonstrates extensive practical application prospects. In recent years, advancements in medical technology and the growing demand for industrial automation have led these technologies to permeate various fields, including minimally invasive surgery, industrial inspection, and micromanipulation (Figure 9A) [127]. This section delves into their specific applications in targeted therapy and drug delivery, cell delivery and biosensing, vascular intervention, and neurosurgical treatment, analyzing and evaluating them based on existing research. For instance, in targeted therapy and drug delivery, magnetic continuums guided by magnetic guidewires enable precise control over drug release locations and timing through external magnetic fields, thereby enhancing therapeutic efficacy and reducing side effects [132,133,134]. A targeted drug delivery system utilizing magnetic nanoparticles efficiently aggregates drugs at tumor sites under magnetic field guidance. Experimental results demonstrate that this technology significantly improves drug delivery efficiency and minimizes damage to healthy tissues. Furthermore, the non-contact nature of magnetically driven systems renders them ideal for cell delivery and biosensor development. In tissue engineering, for example, magnetic continuums guide stem cells or functional cells to specific locations via external magnetic fields, facilitating damaged tissue repair [135]. In the realm of vascular interventions, magnetic continuums are particularly impactful. For example, in percutaneous coronary intervention (PCI), magnetic guidewires can be precisely navigated using magnetic fields to perform accurate operations on narrowed blood vessels, such as balloon dilation or drug release. Compared to traditional methods, this technology notably enhances operational precision and success rates [136]. Regarding neurosurgical treatment, magnetic drive systems are primarily used in cerebrovascular procedures and minimally invasive surgeries. For instance, in atrial fibrillation (AF) ablation, a magnetic catheter guided by a magnetic field allows for precise manipulation of cardiac tissues and completes radiofrequency heating to block abnormal electrical signals [3]. This technique reduces procedural risks and markedly improves therapeutic outcomes.

Despite the considerable potential of magnetic drive systems and magnetic continuums in these areas, several challenges remain. These include achieving more efficient nanoparticle loading, further optimizing magnetic field distribution to enhance operational precision, and ensuring the performance of magnetic materials does not degrade significantly in extreme environments such as high temperatures or humidity [128]. Future research must address these issues to fully realize the clinical and industrial applications of these promising technologies.

Magnetic drive systems and magnetic continuum robotics achieve high-precision operations through non-contact magnetic field actuation, significantly reducing surgical risks and enhancing therapeutic efficacy [137]. Mao et al. integrated functional structures such as micro-cameras, biopsy forceps, and drug-delivery channels to enhance the potential of magnetic continuum robotics for gastric examination and sampling. Additionally, combining ultrasound imaging with magnetic navigation enables effective channel reconstruction and vascular path navigation [2]. Li et al. developed a variable-stiffness concentric magnetic continuum robot (C-MCR), comprising guidewires and concentric tubes with different stiffnesses (Figure 9B) [40]. Due to its adjustable stiffness characteristics, the C-MCR can achieve large curvature bending and has four working modes to accommodate various applications. Experimental results demonstrate that the C-MCR can navigate complex paths through internal channels using a large eight-coil electromagnetic system and deliver nanomedicines over long distances to specific regions, providing a promising application for customized nanomedicine delivery [1]. To minimize radiation exposure to physicians during vascular intervention procedures, Fu et al. developed a magnetically controlled robotic system (MCGRS) (Figure 9C) [115]. Combined with an X-ray imaging system, the MCGRS provides active steering capability via a magnetic actuator system (MAS) and utilizes a guidewire/catheter advancement apparatus (GCA) for precise catheter manipulation. In vivo experiments show that MCGRS successfully accomplished precise navigation and therapeutic access interventions from the femoral artery puncture point to the target vessel. Yang et al. developed a multifunctional variable-stiffness magnetic continuum robot based on phase change material properties (Figure 9D) [128]. The device enables navigation and manipulation of complex channels through coaxially assembled “Guider” and “Follower”. In experiments, the robot navigated through narrow passages, entered open spaces, and successfully navigated to predefined locations in the stomach. It also manipulated the tip of the magnetic continuum to draw shapes on a 2D plane using an external solenoid valve and an air pump system [138]. Moreover, the robot navigates to target locations within the stomach and delivers biopsy forceps along the follower’s working channel, accomplishing biopsy sampling with the support of a distal rigid anchor point established by the follower (Figure 9H) [128]. In vivo bioprinting has emerged as a direct manufacturing technique to fabricate artificial tissues and medical devices at target sites, enabling advanced clinical strategies. Zhou et al. developed a magnetically driven ferromagnetic soft catheter-based robot (FSCR) for minimally invasive in vivo bioprinting (Figure 9E) [139]. By dispersing ferromagnetic particles in a fiber-reinforced polymer matrix and designing inks according to different rheological properties and functional requirements, the system achieved high-precision digitally controlled printing. Its effectiveness was experimentally verified by printing conductive hydrogels on porcine tissue surfaces. Lussi et al. proposed a sub-millimeter continuously variable stiffness catheter (Figure 9F) [51]. The device ensures safe navigation in a soft state and enables rapid stiffness adjustment during manipulation to apply the desired force. The catheter is equipped with magnets and micro-clamps for minimally invasive ophthalmic procedures, assisted by an electromagnetic navigation system. Pancaldi et al. proposed a tethered ultraflexible endovascular microprobe (Figure 9G) to address the lack of effective guidance techniques for magnetic continuums in complex surgical scenarios, such as cerebral vasculature [116]. Driven by hydrokinetic energy and dynamically steered at bifurcations by magnetic head deformation, the device’s cross-sectional area is several orders of magnitude smaller than that of the smallest existing catheters, enabling navigation through tortuous vessels with minimal external intervention. Navigation and manipulation experiments in perfused extracorporeal rabbit ears via the μ-probe verify that the method significantly improves the speed of robot-assisted interventions and enables the simultaneous deployment of multiple leads via standard needle injection and saline perfusion [128]. Wang et al. proposed a soft continuum robot based on thermally stretched microtubules to address challenges in cardiovascular disease treatment (Figure 9H) [127]. Overcoming limitations of conventional magnetic continuums, such as large cross-sectional size, insufficient driving force, and lack of navigation control, the device combines liquid kinetic propulsion and external magnetic field steering strategies. This innovation allows easy navigation within the vasculature and accurate lesion site targeting, expected to enable controlled navigation throughout the vasculature for cardiovascular disease diagnosis and treatment.

This section conducts a systematic analysis of the specific applications of magnetic actuation systems and magnetic continuum robots in the fields of medicine, industrial inspection, and micromanipulation. The results indicate that these technologies offer significant advantages in achieving high-precision non-contact operations. However, there are still some limitations, such as the optimization of magnetic field distribution, the manipulation speed of the system, and the compatibility and stability of materials. Future research needs to further optimize the design and performance of magnetic drive systems and magnetic continuum robots in conjunction with practical application requirements [132,140]. For example, magnetic field responsiveness can be improved by developing new magnetic materials, or adaptive control of complex environments can be realized by introducing artificial intelligence algorithms. Additionally, promoting the application of these technologies in more fields will also be an important research direction. Overall, the research on magnetic drive systems and magnetic continuum robots has broad application prospects. Through continuous technological innovation and optimization, we expect these technologies to revolutionize the fields of medical treatment, industrial inspection, and micromanipulation, ultimately achieving widespread application in clinical and industrial environments.

## 5. Conclusions, Current Challenges and Future Work

This paper reviews the development and application of magnetic control systems and magnetic continuums for medical robots in terms of permanent magnetic and electromagnetic drive systems, magnetic wires and catheters, and variable stiffness magnetic continuums. In terms of technological development, magnetic control systems have encompassed a wide range of types such as permanent magnetic fields (PMFs), electromagnetic fields (EMFs), and commercial systems (e.g., Stereotaxis Niobe platform). Each type exhibits unique advantages and limitations in clinical applications. For example, permanent magnet systems are known for their low energy consumption and high magnetic force, but lack real-time modulation capabilities; EMF systems enable dynamic control, but face limitations in magnetic field strength and heat generation. In addition, magnetic continuum devices (e.g., wires, catheters, and variable-stiffness forceps based on low-melting-point alloys) have shown particular promise for their navigational capabilities and flexibility in complex vascular and neural pathways, opening new possibilities for minimally invasive therapies. Moreover, advances in materials science, fabrication techniques, and control algorithms have greatly improved the performance of these systems. For example, the combination of machine learning and magnetic actuation has optimized navigation accuracy in complex environments, and 3D printing technology has enabled the design of complex structures. Overall, the development and application of magnetically controlled systems and magnetic continua marks an important breakthrough in the field of micro-minimally invasive medicine, providing innovative solutions for complex surgical interventions and treatments. Through the combination of magnetic actuation and continuum structures, non-contact manipulation, real-time adaptability, and greater operational safety are realized, advancing the feasibility of precision medicine.

Despite a series of advances, multiple challenges still need to be overcome to achieve widespread clinical applications: first, the optimization of magnetic field distribution is crucial as it is directly related to operational precision and efficiency; second, the lack of reliability of magnetic materials in high-temperature or humid environments restricts their application in extreme conditions; third, the design and fabrication of magnetic continuums require a balance between flexibility, rigidity, and susceptibility to external magnetic field balance between flexibility, rigidity, and sensitivity to external magnetic fields, which increases manufacturing complexity and cost. For example, the high price of commercial systems makes it difficult to generalize them to resource-limited environments; furthermore, the lack of standardized preclinical testing and validation protocols hinders the large-scale application of these devices.

To address the core challenges of the current magnetron continuum system, future research directions can start from the following aspects: firstly, constructing an intelligent magnetic field regulation system by integrating deep reinforcement learning with coupled multi-physics field modeling, and combining the magnetic field database of clinical scenarios in order to optimize the operation accuracy. Moreover, the introduction of artificial intelligence (AI) and machine learning (ML) algorithms will play an important role in optimizing the navigation accuracy to improve the intelligence of the system by adapting to the dynamic conditions and predicting the best path in real time. Secondly, the development of new magnetic materials is key, especially those with enhanced responsiveness, durability and phase change properties, which will significantly improve the performance of the devices. For example, designs based on nanoparticles or composites may offer new solutions for magnetic field response. Third, optimizing the structural design and fabrication feasibility of the magnetic continuum, incorporating practical fabrication techniques and engineering production requirements while meeting operational conditions, makes the research feasible for clinical validation. Alternatively, from the perspective of multi-field drive manipulation, utilizing the advantages of independent field decoupling drive to improve the structural design, thus reducing the manufacturing difficulty. And, the development of hybrid drive systems (e.g., combining magnetic drive with hydrodynamic or electric field propulsion modes) will greatly expand the application areas and provide more possibilities for operation in complex environments. Fourth, AI-driven predictive modeling could reduce the need for animal testing and speed up the preclinical testing process. Finally, reducing device size while maintaining functionality will be an important trend, with technologies such as innovative coil designs, lightweight materials, and energy-efficient power supplies enabling researchers to realize more compact and portable systems. Interdisciplinary research will also be an important driver in this area, integrating expertise from a number of fields such as materials science, robotics, artificial intelligence, and clinical medicine will accelerate the translation process from lab to clinical application.

Overall, magnetron systems and magnetic continuums represent a major breakthrough in medical robotics, offering unprecedented precision and safety for minimally invasive therapies. Although many challenges remain, through continued research innovation and technological breakthroughs, this field is expected to overcome existing limitations and drive transformative developments in healthcare. In the future, with the further integration of novel materials, artificial intelligence, and multi-physics field simulation, magnetron systems will be applied in a wider range of clinical scenarios, ultimately maximizing patient outcomes and reducing treatment risks. Continued advances in this field will not only drive technological innovation in minimally invasive therapies, but also lay a solid foundation for personalized medicine and precision medicine in the future.

## Figures and Tables

**Table 1:** Comparative analysis of magnetic drive system characteristics.

Type	Strength	Manipulation	Scenes	Disadvantages	Reference
PMF	Strong	Robotic arm programming	Open Run Scenarios	Safety of magnetic field arrays	[103]
EMF	Weak	Current Signal Programming	Coil surround area	Weak field strength, little operating space	[104]
CMF	Medium	Electromagnetic and permanent magnet systems work together	Semi-open operating table	Control algorithms for permanent–electromagnetic hybrid drive systems	[105]

**Table 2:** Comparative characterization of conventional magnetic wire and catheter, with variable stiffness magnetic continuum.

Type	Stiffness	Scenes	Advantages	Disadvantages	Reference
MW	Low	Cardiovascular stenosis or curvature	Flexibility and mobility	Simple functionality, unable to realize complex operations	[131]
MC	High	Digestive gastrointestinal area	Support, conveying capacity	Prone to jamming and compression of lumen	[40]
VSM	Variable	Vascular and digestive	Shaping and precise manipulation	Complex design, preparation and assembly of variable stiffness layers	[102]

## Data Availability

No data were generated or used during the study.
